# Perspective on Regional Sea-level Change and Coastal Impacts

**DOI:** 10.1017/cft.2024.15

**Published:** 2024-11-12

**Authors:** Kathleen L. McInnes, Robert J. Nicholls, Roderik van de Wal, David Behar, Ivan D. Haigh, Benjamin D. Hamlington, Jochen Hinkel, Daniella Hirschfeld, Benjamin P. Horton, Angelique Melet, Matthew D. Palmer, Alexander A. Robel, Detlef Stammer, Abby Sullivan

**Affiliations:** 1 Climate Science Centre, CSIRO Environment, Aspendale, 3195, Australia; 2 Tyndall Centre for Climate Change Research, University of East Anglia, Norwich, UK; 3 Institute for Marine and Atmospheric Research Utrecht, Utrecht University, Utrecht, The Netherlands; 4 Department of Physical Geography, Utrecht University, Utrecht, The Netherlands; 5 San Francisco Public Utilities Commission, San Francisco, CA, USA; 6 School of Ocean and Earth Science, University of Southampton, National Oceanography Centre, Southampton, UK; 7 Jet Propulsion Laboratory, California Institute of Technology, Pasadena, CA, USA; 8 Global Climate Forum (GCF), Berlin, Germany; 9 Division of Resource Economics, Albrecht Daniel Thaer‐Institute and Berlin Workshop in Institutional Analysis of Social‐Ecological Systems (WINS), Humboldt‐University, Berlin, Germany; 10 Department of Landscape Architecture and Environmental Planning, Utah State University, 4005 Old Main Hill, Logan, UT 84322-4005, USA; 11 Earth Observatory of Singapore, Nanyang Technological University, Singapore; 12 Asian School of the Environment, Nanyang Technological University, Singapore; 13 Mercator Ocean International, Toulouse, France; 14 Met Office, FitzRoy Road, Exeter, EX1 3 PB, United Kingdom; 15 University of Bristol, Bristol, BS8 1UH, United Kingdom; 16 School of Earth and Atmospheric Sciences, Georgia Institute of Technology, Atlanta, GA 30318, USA; 17 Centrum für Erdsystemforschung und Nachhaltigkeit, Universität Hamburg, Hamburg, Germany; 18 City of Philadelphia, Offices of Sustainability and Climate Resilience, 1515 Arch Street, Philadelphia, PA 19102, USA.

## Abstract

We synthesize sea-level science developments, priorities and practitioner needs at the end of the 10-year World Climate Research Program Grand Challenge ’Regional Sea-Level Change and Coastal Impacts’. Sea-level science and associated climate services have progressed but are unevenly distributed. There remains deep uncertainty concerning high-end and long-term sea-level projections due to indeterminate emissions, the ice sheet response and other climate tipping points. These are priorities for sea-level science. At the same time practitioners need climate services that provide localized information including median and curated high-end sea-level projections for long-term planning, together with information to address near-term pressures, including extreme sea level-related hazards and land subsidence, which can greatly exceed current rates of climate-induced sea-level rise in some populous coastal settlements. To maximise the impact of scientific knowledge, ongoing co-production between science and practitioner communities is essential. Here we report on recent progress and ways forward for the next decade.

## Impact statement

Rising sea levels are a major concern for low-lying coastal communities and ecosystems across the globe, yet planning for future sea-level rise is hampered by uncertainties in future greenhouse gas emissions, how ice sheets will respond and other potential climate tipping points that lead to a wide range of possible future projections. The World Climate Research Program Grand Challenge on ’Regional Sea-Level Change and Coastal Impacts’ was implemented to further advance understanding of natural and human contributions to sea-level rise, promote advances in observations and foster the development of sea-level information that assists coastal practitioners in planning for the future. Priority sea-level information for coastal practitioners includes both mid-range and high-end sea-level projections for future planning as well as information to assist with near-term planning. This includes information on extreme sea-levels and associated hazards and land subsidence, which, in some coastal locations, greatly exceeds current rates of climate-induced sea-level rise. This article summarizes recent progress and ways forward for the next decade.

## Introduction

**Figure 1.**
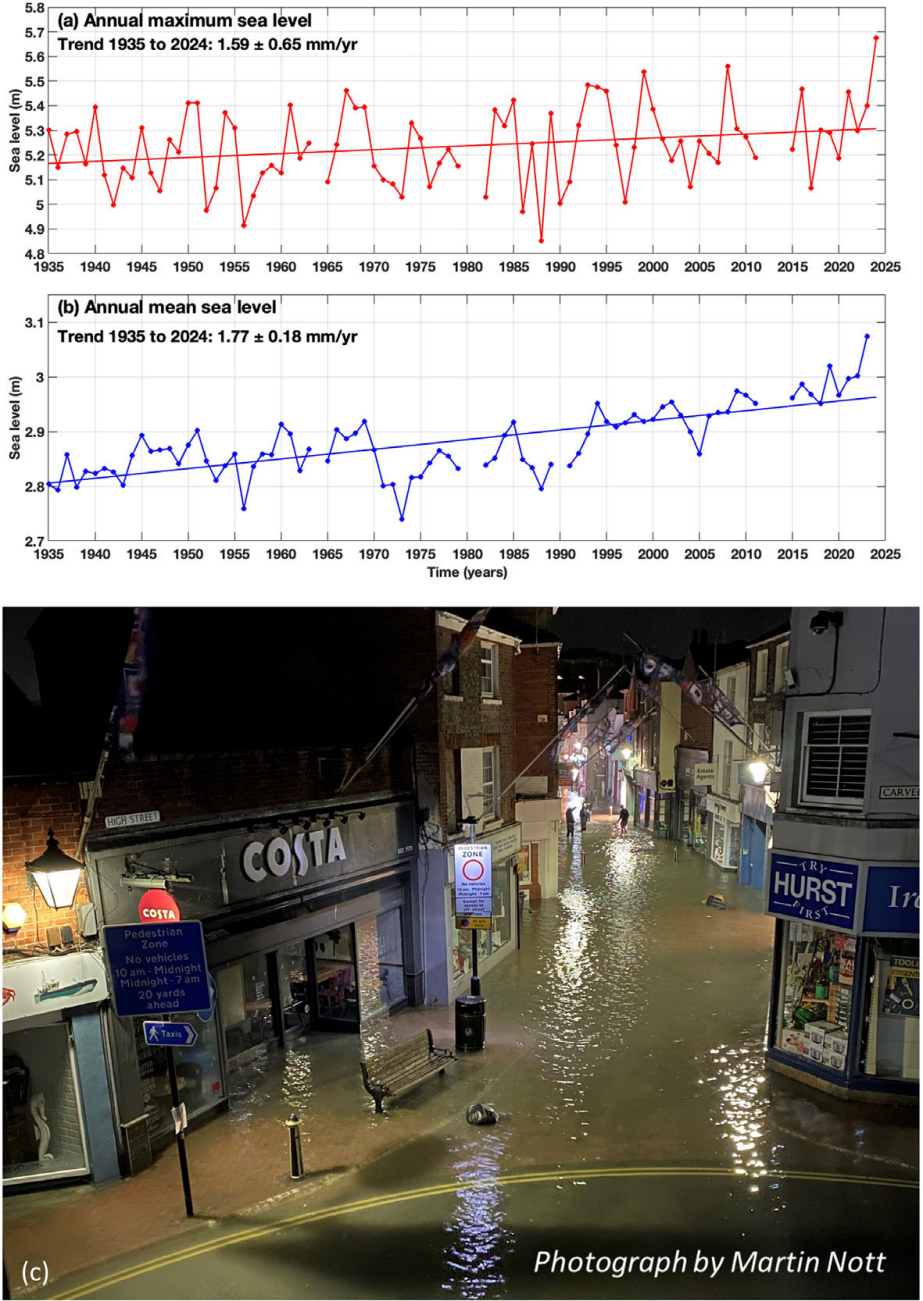
Rising sea levels are raising extreme sea levels and exacerbating floods around most of the world’s coasts. Trends in (a) annual maximum and (b) annual-mean sea level at Southampton, UK from tide gauge data since 1935 showing a rising trend for both and the highest extreme occurring in 2024 (8/9 April). On this night, coastal floods occurred along about 100 km of the central English Channel coast, including (c) the High Street in Cowes, Isle of Wight, UK.

To meet urgent societal needs for useful information on sea-level rise (SLR), the World Climate Research Program (WCRP) implemented the theme ’Regional Sea-Level Change and Coastal Impacts’ as one of its cross-cutting science questions, or Grand Challenges (GC). The GC objectives were to: (1) establish a quantitative understanding of the natural and anthropogenic mechanisms of regional to local sea-level change and variability; (2) promote advances in observing systems required for integrated sea-level monitoring; and (3) foster the development of sea level information to further benefit coastal zone managers, who are dealing with the consequences of rising mean and extreme sea levels (Figure 1). An interdisciplinary program was developed encompassing global to regional and local scales. In particular, the program aimed for close interaction with relevant coastal stakeholders to increase the utility of scientific research for coastal zone management, and impacts and adaptation efforts. The program entailed work on paleo-timescale sea-level estimates, land-ice contributions to SLR, regional sea-level variability and change including extremes, regional sea-level predictability, sea-level science for coastal zone management, sea-level budget, and identification of practitioner needs from climate science through practitioner engagement.

The GC facilitated many important publications. These include the identification of users’ needs for SLR information, including high-end SLR projections, in decision making (Hinkel et al., 2019), a transparent framework for developing high-end SLR projections (Stammer et al., 2019) subsequently applied to 2100 and 2300 for a low and high emission scenario in van de Wal et al., (2022). Sea-level variability and change on various spatiotemporal scales were the topics of a workshop and journal special edition (Ponte et al., 2019b and references therein), including a paper highlighting the large uncertainties associated with projected Antarctic mass loss (van de Wal et al., 2019). A consistent terminology for the sea level community, including vertical reference frames, SLR components and extremes, was addressed in Gregory et al., (2019). An international collaboration to assemble and assess the data quality of SLR sources, allowed estimates of land-based ice and thermal expansion over 1993-2018 to be refined, but uncertainty in the land water storage component remains (WCRP Global Sea Level Budget Group 2018).

Ponte et al., (2019a) reviewed observational platforms and modelling systems for simulating and predicting coastal sea level. A review of the status of coastal services to deliver sea level information in Le Cozannet et al., (2017), was followed by a dedicated workshop and a special journal edition on the topic (Le Cozannet et al., 2022 and references therein). Linked work evaluated the significance of subsidence in coastal cities and deltas, which demonstrated the prevalence of coastal residents in subsiding areas which, on average, experience relative SLR up to four times faster than that due to climate change alone (when weighted by population) highlighting the urgency of effective coastal adaptation (Nicholls et al., 2021). The GC also undertook the first global survey of coastal practitioners to understand whether and how SLR projections were being used and to ascertain other information practitioners require for coastal adaptation decision making (Hirschfeld et al., 2023).

Three significant international sea-level conferences and workshops were organised by GC members; an initial conference in New York in 2017, practitioner-led workshops in 2022, and the final (sunset) conference in Singapore, later in 2022. The 2017 conference highlighted research priorities that shaped GC activities in subsequent years. The need for stronger engagement with the practitioner community was identified as critical for providing salient information for future adaptation. The practitioner-led workshops, in turn, included identification of gaps and needs in the production and translation of climate science to support coastal resilience planning (see section 4.1). The final GC conference in Singapore enabled assessment of progress since 2017 and had a more prominent practitioner focus. Both the workshops and the Singapore gathering contributed to the launch of a global community of practice focused on coastal resilience, the Practitioner Exchange for Effective Response to Sea Level Rise (PEERS, www.peerscoastal.org).

In this article, which will serve as a legacy of the work of the GC, we take stock of the major advancements in sea level science over the past decade. We draw on presentations and discussions from the final Singapore conference to provide a perspective of the topics that continue to require urgent attention, particularly as we begin the Intergovernmental Panel for Climate Change (IPCC) seventh assessment cycle. In the remainder of this article, we address in more detail advances in data supporting sea level science (section 2), sea level science advances (section 3), practitioner perspectives and needs (section 4) and future priorities (section 5).

## Sea-level observations and evidence from past climates

### In-situ and satellite observations

**Figure 2.**
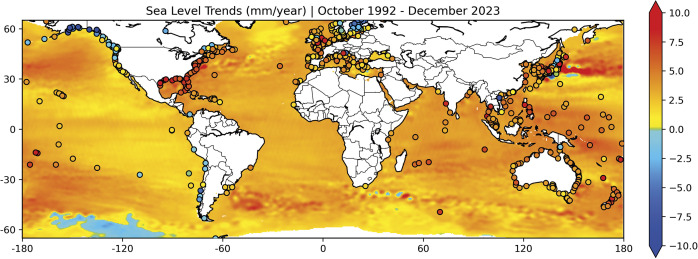
Coastal and regional sea level trends (mm/yr) over October 1992-December 2023 (31-yr time span) from reprocessed Jason-1, 2 and 3 missions. Trends at tide gauges with at least 20 years data within the altimeter time period, sourced from the Permanent Service for Mean Sea Level are shown by circles, noting that tide gauge trends are calculated over the available time period. The background map shows regional sea level trends from the NASA SSH Gridded Dataset (Fournier et al., 2024).

Over the past decade, sea level observations have been sustained and improved. The launch of Sentinel-6A in 2020, sees the record of high precision, near-global sea-level measurements from conventional radar satellite altimetry now exceeding three decades (Donlon et al., 2021; Hamlington et al., 2023). The continuous record of this reference mission, supported by several satellites, has led not only to definitive estimates of rising regional and global sea levels, but also the increasing rate of global SLR (e.g., Nerem et al., 2018; Guérou et al., 2023). Overall accuracy has improved from one satellite to the next and improved technology and advanced processing approaches have led to better measurements of smaller scales of sea level variability, which now also extend closer to the coasts. The latter is particularly important for risk and adaptation assessments. During the GC, new missions in coastal altimetry (e.g., Cipollini et al., 2017, Birol et al., 2017, Vignudelli et al., 2019) and/or waveform retracking (e.g., Passaro et al., 2014, Birol et al., 2021) progressed substantially, enabling analysis of decadal coastal sea level trends (Cazenave et al. 2022). The large regional variations in SLR trends are illustrated in Figure 2.

In 2022, a breakthrough in satellite altimetry occurred with the launch of the Surface Water and Ocean Topography (SWOT) mission (Morrow et al., 2019). SWOT uses radar interferometry to measure ocean and surface water levels over a 120-km wide swath with a roughly 20-km gap along the nadir that is partially filled by a conventional radar altimeter. The orbit repeats every 21 days, but the swath measurements result in much of the globe having a revisit time that is significantly shorter while also filling in many of the gaps in the current observations of sea level. Initial analyses indicate a dramatic improvement in spatial resolution of sea-level data, including observations up to and through the coastal interface. In addition, other satellites have contributed to an increasingly dense network of higher resolution, altimeter measurements in polar regions in the past decade, including Cryosat-2, with a synthetic aperture interferometric radar altimeter (Wingham et al., 2006), Sentinel-3A, -3B (Clerc et al., 2020), Sentinel-6A with synthetic aperture radars, and ICESat-2 with a laser altimeter (Neumann et al., 2019).

Observations of ocean temperature and salinity profiles have increased and improved through increased numbers of ARGO floats and corrections of instrumental biases (Boyer et al., 2016). Gravimetry for ocean mass changes - barystatic sea-level changes (GRACE and GRACE-FO, Landerer et al., 2020) have also progressed. This has enabled improved understanding of SLR and the closure of the observed SLR budget at global (Fox-Kemper et al., 2021) and regional scales (Dangendorf et al., 2021; Marcos et al., 2019; Frederikse et al., 2020; Camargo et al., 2023), at least until 2016 (Nerem et al. 2018; WCRP Global Sea Level Budget Group, 2018).

For longer time scales, tide gauges are the major source of coastal SL observations monitoring most of the world coastlines (e.g., Marcos et al., 2019). The Permanent Service for Mean Sea Level (PSMSL), which was established in 1933, has been responsible for the collection of mean sea-level data from global tide gauges (Holgate et al., 2012) and produces monthly and annual mean sea level datasets. These have been used, with altimeter records, in most past mean sea-level trend and variability studies. The Global Extreme Sea Level Analysis (GESLA) provides high-frequency (at least hourly) sea-level information from tide gauge stations distributed worldwide. The first GESLA dataset was compiled in 2009, with a second update in 2015/16 (Woodworth et al., 2016) and a major third update in 2022/23, with the dataset currently comprising 91,021 years of data from 5,119 records (Haigh et al., 2023). The Joint Archive for Sea Level (Caldwell et al., 2015), established in 1987 and hosted by the University of Hawaii Sea Level Center (UHSLC), forms an important part of the GESLA dataset. For higher-frequency monitoring required for studying oceanographic processes like seiches, meteotsunamis, infragravity, and coastal waves, a 1-min SL dataset (Minute Sea-Level Analysis, MISELA) was developed at 331 tide gauges worldwide (Zemunik et al., 2021).

### Synthesis Data Programmes

Several data programmes have been developed over the last decade to synthesize sea-level changes. The European Union’s Earth Observation Programme, Copernicus, provides information on sea-level changes through in-situ datasets, satellite observations (including from Sentinel missions), ocean reanalyses covering the past decades and near-term forecasts. Copernicus also provides ancillary fields needed to assess SLR-induced coastal risks (coastal land cover and land use, vertical land motion, digital elevation models, flood monitoring, etc.), to guide adaptation and support related policies and directives (see Melet et al., 2021). In addition to ongoing dataset improvements, Copernicus Services plan to improve their SLR products and services and associated risks through the addition of time-evolving satellite-derived coastal bathymetry and shoreline position, continuous monitoring of coastal floods, provision of longer-term past sea-level changes (i.e. extended reanalyses) and regionalized future climate projections (e.g., Chaigneau et al., 2022), attribution of extremes, and mapping of coastal defense structures across Europe’s coasts (Melet et al., 2021). A web platform, the Copernicus Coastal Hub, has been developed to provide the relevant core services of Copernicus to end-users.

Separately, the NASA Sea-Level Change Team has worked to both improve the understanding of sea-level change in the past and future through interdisciplinary research and to strengthening the connection to practitioners and end-users with the goal of advancing access to global sea-level data and information. This includes, for example, establishing partnerships with the IPCC to deliver the updated sea-level projections from the recent 6th Assessment Report (AR6; Fox-Kemper et al., 2021; https://sealevel.nasa.gov/ipcc-ar6-sea-level-projection-tool). Dedicated efforts to engage and support practitioners are ongoing, as are efforts to synthesize and integrate disparate Earth observations into improved information on sea-level change.

### On the use of paleodata

The ice sheets, oceans and the solid Earth are the Earth system components that change most slowly under climate change. Consequently, the rapid changes since pre-industrial times are not in equilibrium with the current forcing of the climate system. One of the major challenges in ice sheet modelling is therefore to capture this imbalance. One option is to use observations of sea-level rates and high-stands in the warmer past (e.g. Eemian). A full understanding of Eemian high-stands is still missing as the contribution from Antarctica is poorly constrained for slightly warmer conditions than present-day, mainly due to a lack of understanding of the ice-ocean interaction, but also because the magnitude of the high-stand is also strongly dependent on the assumptions made to estimate the Glacial Isostatic Adjustment (Dyer et al., 2021). The physics behind basal melt is also important for explaining current rates of mass loss in West-Antarctica. The aim is that the physical processes constrained by modern and geological observations can be captured adequately. However, few studies have attempted to use paleo sea-level information to project SLR in future. Notable is the work by DeConto et al., (2021) where geological observations constrain model parameters, especially those controlling marine ice-cliff instability.

A further application of paleo data, important for stakeholders, is whether and when sea level started to accelerate. Sea-level reconstructions of the Common Era (last 2000 years) have been used to estimate the timing of the acceleration or inception of modern rates of SLR, since they extend the instrumental record back before the 20th century and have improved attribution of sea-level change (e.g. Kemp et al., 2011). Walker et al., (2022) used a global database of proxy sea-level records of the Common Era to show that globally, it is very likely that rates of SLR emerged above pre-industrial rates by 1863 CE (P = 0.9; range of 1825 [P = 0.66] to 1873 CE [P = 0.95]), which is similar in timing to evidence for early ocean warming and glacier melt, which caused most SLR over the 20th century.

## Modelling and projections of sea-level change

### State of the Art Sea Level Projections

Sea-level projections based on process models involve combining the contributions of ocean dynamic sea level from the Coupled Model Intercomparison Project (CMIP) climate models, run to support the IPCC process, with other components of sea-level change. These include terrestrial water storage changes, Glacial Isostatic Adjustment (GIA), spatial redistribution of sea level due to gravitational, rotational and deformational changes in the Earth in response to ice-sheet mass changes (sometimes referred to as sea-level fingerprints) and the SLR from dynamical processes that contribute to ice sheet and glacier mass loss, which are obtained from separate off-line models usually forced by output from CMIP climate models, thereby implicitly ignoring feedbacks between climate and ice sheet models.

**Figure 3.**
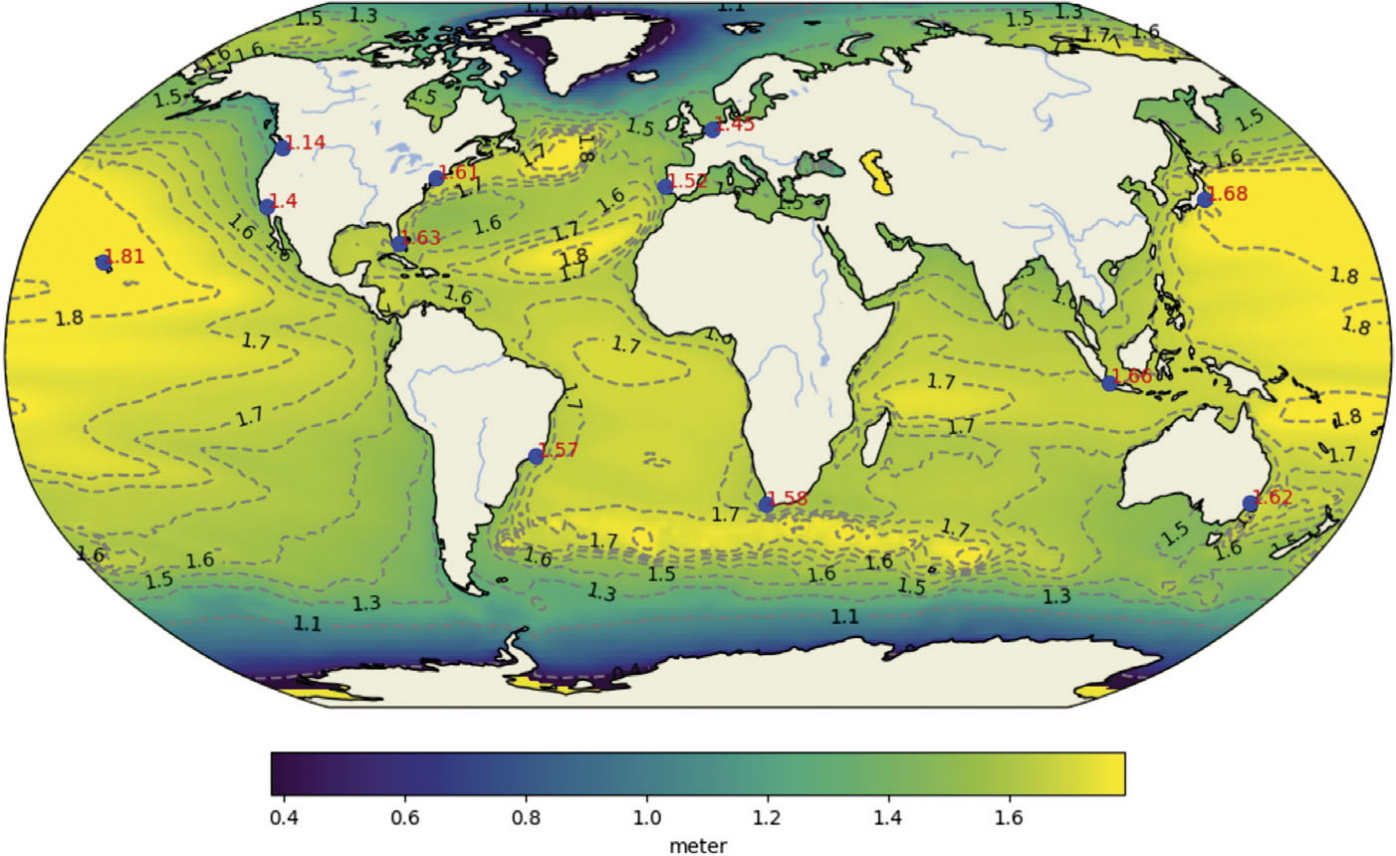
Regional high-end SLR projections based on van de Wal et al., (2022), for 2100 under RCP8.5/SSP5-8.5, with a global-averaged value of 1.55 m. Numeric values of SLR are provided for Honolulu, Seattle, Los Angeles, New York, Miami, Rio de Janeiro, Den Haag, Lisbon, Cape Town, Jakarta, Tokyo and Sydney.

State-of-the-art sea-level projections presented in the IPCC Sixth Assessment Report of Working Group I (AR6; Fox-Kemper et al., 2021) incorporated several methodological advancements relative to IPCC AR5 (Church et al., 2013) and the IPCC Special Report on Oceans and Climate Change (SROCC; Oppenheimer et al., 2019. These included: (i) use of physically-based emulators, which allowed for sea-level projections that were consistent with the AR6 assessment of climate sensitivity (Forster et al., 2021) and also consistent inclusion of ice sheet modelling from previous assessments (Slangen et al., 2023); and (ii) use of coordinated community process-modelling efforts for the ice sheets (Goelzer et al., 2020, Levermann et al., 2020; Seroussi et al., 2020) and glacier response under climate change (Marzeion et al., 2020). Despite these advances, the ‘likely range’ projections, which characterize the central two-thirds of the probability distribution, remain broadly similar across AR5, SROCC and AR6 (Slangen et al., 2023). However, high-end sea-level change, caused by poorly understood physical processes inducing ice-mass loss of the Antarctic ice sheet, is uncertain. There is a low confidence, high-impact storyline based on expert elicitation and exploratory modeling presented in AR6 that could exceed 2 m of GMSL rise by 2100 and 15 m by 2300. More recently, an analysis emerging from the GC based on physical storylines arrived at lower values for both 2100 (1.27-1.55 m) and 2300 (up to 10 m) (Van de Wal et al., 2022). Figure 3 presents the estimated regional high-end values following the approach by Van de Wal et al., (2022).

On even longer time scales, Turner et al., (2023) developed SLR projections to 2500 that help to illustrate the multi-century commitment and long-term benefits of mitigation action. Similarly, Palmer et al., (2024) developed multi-century SLR projections in a flexible storyline framework that can be tailored to stakeholder needs or specific decision-making contexts.

### Advances in ice sheet modelling

To constrain the low likelihood probabilities of SLR it is critical to develop ice models further. Most ice sheet models used for SLR projections still compare poorly to observations of ice sheet change over the last 20 years (Aschwanden et al., 2021) and during past warm periods (Dutton et al., 2015). However, through advances in model representation of processes occurring at the boundaries of ice sheets and model architecture, some individual models have greatly improved their fidelity to past observed changes (Nias et al., 2016, DeConto et al., 2021, Golledge et al., 2019, Gilford et al., 2020).

Surface mass balance models have improved in modeling firn compaction and water retention within snow (e.g. Lundin et al. 2017), and Earth System Models (ESMs) are performing better for Greenland. Similarly, simulated ocean melting of ice sheets has improved in contemporary models (Cowton et al. 2019, Lambert et al. 2023), but still disagrees with observations, particularly where ocean circulation interacts with subglacial discharge. Models which include ocean intrusion and melting underneath grounded ice sheets predict nearly twice the rate of future SLR (Seroussi et. al. 2019, Robel et al., 2022). Other models of rapid iceberg calving at tall ice cliffs (Bassis and Walker 2012; Crawford et al., 2021) have suggested the possibility of even higher future SLR (DeConto et al., 2021), though other calving models suggest such rapid calving states may be ephemeral (Clerc et al., 2019, Bassis et al., 2021). At the ice sheet base, models of glacial isostatic adjustment, gravitationally self-consistent sea level (Gomez et al., 2018, van Calcar et al., 2023) and subglacial hydrology (Schoof et al., 2010) have also raised the possibility of new negative feedbacks on future ice loss from both Antarctica and Greenland.

Several modeling centers have focused efforts on coupling ice sheet models with oceanic and atmospheric models or incorporating them fully into ESMs (Smith et al., 2021). Decades of progress in transient data assimilation in the weather and climate modeling communities are now translating to rapid improvements in the way ice sheet models are being initialized and calibrated (e.g., Goldberg et al., 2022, Van den Akker et al., 2024). Additionally, many ice sheet models now incorporate stochastic and neural-network parameterizations (Jouvet et al., 2022, Verjans et al., 2022, Ultee et al., 2023) to improve their speed and ensemble capabilities for better uncertainty quantification. All these ice-sheet model improvements will facilitate coupling in ESMs and better calibration with present-day observed environmental conditions, improving SLR projections mostly for the near future.

### From Regional SLR to local projections of coastal hazards

Coastal adaptation requires SLR projections that are tailored to local conditions together with additional information on extreme coastal sea levels from which coastal hazards (e.g. flooding and erosion) may be calculated. While CMIP models provide information on local SLR changes due to ocean density and circulation, the typical 1° spatial resolution of the ocean models means they are unable to resolve complex circulations along continental shelves (Zhang et al., 2016; Van Westen and Dijkstra, 2021). The application of higher resolution global (Zhang et al., 2017; Jin et al., 2024) or regional ocean models (e.g. Toste et al., 2018; Hermans et al., 2021; Jin et al., 2021; Shin and Alexander, 2020; Chaigneau et al., 2022) is enabling improved representation of ocean circulation and better resolved dynamic SLR projections closer to the coast.

Coastal hazard assessments require information on sea-level extremes that consider astronomical tides, weather-induced storm surges and wind-waves (infragravity waves, wave setup, wave runup), their associated uncertainties expressed as probabilities of occurrence over different time periods and accurate digital elevation models (Hinkel et al., 2021). Advances have been made with global-scale hydrodynamic models of tides and storm surge (e.g. Muis et al., 2016, 2020), and tide-surge-waves (e.g., Mentaschi et al., 2023), with forcing provided by meteorological reanalysis (e.g. Dullaart et al., 2020) and tropical cyclones (Bloemendaal et al., 2019). Coordinated efforts are underway to progress global modelling efforts (Bernier et al., 2024). The combination of data from storm surges and tide models with wave setup derived from wave model reanalyses has enabled the derivation of extreme sea-level statistics for use in global coastal flood assessments (e.g. Rueda et al., 2017; Vousdoukas et al., 2018; Kirezci et al., 2020).

Although there have been advances in large scale assessments of coastal hazards, stakeholders often need localized information that may be limited or unavailable, such as elevation, bathymetry, vertical land movement (e.g. Nicholls et al., 2021) and river flows. Furthermore, the coincidence of high river flows and/or intense precipitation events with extreme coastal sea levels can cause compound flooding (e.g. Wahl et al., 2015, Bevacqua et al., 2019, Collins et al., 2019, Bevacqua et al., 2020, Couasnon et al., 2020, Hermans et al., 2024). Green et al., (2024) recently provided a comprehensive review of compound flooding in coastal regions. Establishing the probabilities of extreme sea levels from all contributing factors under present and future climate conditions is a major computational undertaking. To address this complexity, hybrid statistical-dynamical approaches akin to machine learning methods are being developed to estimate nearshore coastal hazards (e.g. Camus et al., 2014; Cagigal et al., 2020, Ayyad et al., 2023).

## Engaging with the practitioner perspective

The GC considered practitioner and decision-making perspectives to facilitate use of the science results summarized in Sections 2 and 3. The main focus here is on risk assessment and adaptation decisions up to a century in the future, reflecting the practitioner needs that were expressed in the GC around practical action.

### Challenges practitioners are facing

Preparing for SLR requires practitioners to understand the magnitude and rate of change, associated uncertainties, their local implications, and the societal context in which decisions are made. Practitioners bring relevant expertise, including local regulations and permitting processes, funding options, stakeholder perspectives including local politics, but generally lack time to follow evolving climate science. Therefore, no global standard in the uptake of SLR projections into planning exists and practitioner approaches vary widely (Hirschfeld et al., 2023). The myriad coastal hazards associated with SLR (erosion, flooding, salinisation, etc.) further complicate practitioners’ analysis. To better understand these issues, two global workshops were convened to share knowledge among practitioners on how SLR science is incorporated into decision-making, understand the state of coastal adaptation planning and action, and address communicating the case for action (Boyle et al., 2022; Hirschfeld et al., 2024). Lessons were shared at the WCRP GC SLR conference in Singapore (2022) and are summarized below.

#### Challenges working with observations and projections

**Figure 4.**
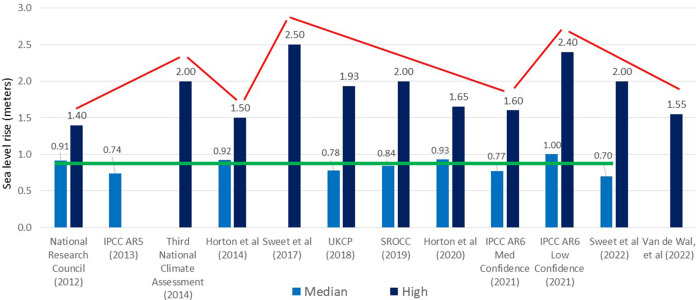
Median and high-end SLR scenarios for the year 2100, produced by papers from 2012 to 2022. The high-end scenarios show large fluctuation compared to the median (see Supplemental Information for further details). Note the references for the above-mentioned reports: National Research Council (2012) -Dalrymple et al., (2012); Third National Climate Assessment, (2014) – Melillo et al., (2014); IPCC AR5 – Church et al., (2013); UKCP (2018) – Palmer et al., 2018; SROCC (2019) – Oppenheimer et al., (2019); IPCC AR6 (2021) – Fox-Kemper et al., (2021).

Many practitioners lack access to relevant local observations or downscaled SLR projections with the Southern hemisphere and developing countries most deficient. The scientific literature requires translation by climate service providers or boundary workers (also referred to as knowledge brokers; Lomas, 2007; Harvey et al., 2012) working with practitioners to characterize knowledge and uncertainty into actionable information. This is particularly true for long-term high-end SLR projections, which are important for risk management (Hinkel et al., 2015; 2019) and attract strong practitioner attention. Recent high-end projections have caused confusion among practitioners (Boyle et al 2022), as authoritative sources published over the last 11 years have fluctuated by a meter or more at the high end, while median SLR projections remained relatively constant (Figure 4). A compounding issue is that the speed of planning/implementation - approximately two or three decades for major capital projects – is much slower than the release and adoption of new high-end projections in influential forums (Figure 4; Boyle et al., 2022; Lipscomb et al., 2024). This emphasizes the need for actionable science as discussed below.

#### Barriers to understanding and communicating impacts

Beyond mean and high-end SLR projections, practitioners need to assess other related coastal information and hazards (Section 3.3), such as subsidence, sea-level extremes, erosion, saltwater intrusion and more. Compound threats are costly and difficult to assess and there is a gap between the science discussed earlier and the availability of localized compound information. Many practitioners lack access to high-resolution inundation models, which are a valuable visualization tool to communicate risk (Boyle et al., 2022). This widens the gap between places that are adapting and those that cannot. Despite facing existential risk, many small islands appear in the latter category. One partnership addressing this gap is a PEERS/NASA effort to coproduce inundation maps (https://peerscoastal.org/get-involved/inundation-mapping).

### Elements for addressing practitioner challenges

**Figure 5.**
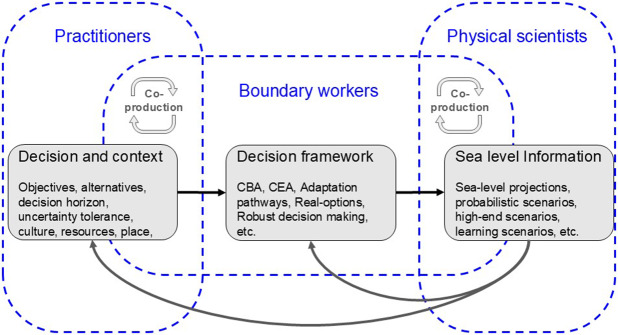
The three core steps of an idealised iterative workflow of developing actionable sea-level information (black boxes) and roles of the individuals involved (blue dashed boxes; practitioners, boundary workers and sea-level scientists). Boundary workers are involved in all three steps but with a focus on the decision making framework connecting the work of sea-level scientists and practitioners (CBA=Cost-benefit analysis, CEA=Cost-effectiveness analysis).

The following needs were identified over the course of the GC:

- **Co-Production, robust climate services and boundary support** - Increased collaboration between practitioners, boundary workers and climate scientists to co-produce knowledge was affirmed as essential to advancing global adaptation (Figure 5). Boundary workers play a critical role in the translation between practitioners and scientists, but climate services are poorly developed for coasts (Le Cozannet et al., 2017; 2022), hindering progress.
- **Development of actionable science** - That is, science that is widely agreed upon in the scientific community (Bamzai-Dodson et al., 2021; Lipscomb et al., 2024). While IPCC reports in recent years have expanded the type of SLR projections to assist with risk assessment (e.g. SROCC, AR6), an unintended consequence has been to raise the profile of uncertain, experimental outputs not yet replicated by the broader scientific community without providing sufficient guidance for practitioner uptake. GC initiatives led to Stammer et al., (2019) and Van de Wal et al., (2022), which directly addressed practitioner needs by accentuating high-end SLR projections supported by multiple lines of evidence, transparency, and scientific confidence. Building on this work, an actionable science definition has been proposed: “*A scientific claim is sufficiently accepted to justify adaptation action (i.e., near-term physical measures and financial investments) when it is supported by multiple, consistent independent lines of high-quality evidence leading to high or medium confidence, as determined by a diverse group of experts in an open, transparent process”.* (Lipscomb et al, 2024). Efforts to develop consistent, clear approaches for translating SLR science into actionable information to underpin adaptation investment are needed, ideally featuring coproduction partnerships between practitioners and scientists.
- **Development of a community of practice -** Needed to support practitioners developing leading practices in adaptation. PEERS was established in 2023 by participants of the global workshop and Singapore conference and at this writing has over 500 members in 59 countries with strong global North and global South participation.

#### Co-production and boundary support

The GC developed a framework to facilitate the production of SLR information to meet practitioner needs (Hinkel et al., 2019). This starts with the practitioner’s decisions and associated context. These differ from case to case and require diverse decision-making frameworks and types of SLR information. The decision context includes: (1) the uncertainty tolerance; a low uncertainty tolerance equating to the preparation for unlikely but extreme outcomes; (2) the decision or time horizon, for planning, implementation and operation of the adaptation measures -- ranging from years (e.g., beach nourishment), to decades (e.g., protection infrastructure such as dikes, land reclamation in small islands), to a century or more (e.g., critical infrastructure such as nuclear power, long-term land-use planning) (Burcharth et al., 2014; Rigo et al., 2006; Hino et al., 2017; Wilby et al., 2011; Hinkel et al., 2023); and (3) the ability to adaptively manage the response, which is most relevant for long-term adaptation.

Three additional contextual aspects emerged in the practitioner workshops: culture, resources, and place (Hirschfeld et. al., 2024). Culture shapes how practitioners think and thus influences their needs (e.g., attitudes towards protection versus the environment), their uncertainty tolerance and decision horizons. Human, natural, and financial resources, or lack of resources, all influence a practitioner’s requirements from boundary scientists (Aylett, 2015). Practitioners also consider different physical attributes of places (e.g., topography, tidal range, etc.) and people (i.e., high density, medium density, etc.) influencing exposure and vulnerability and the information required.

The practitioner workshops identified that co-production between SLR scientists, practitioners and boundary workers is essential (see also Hewitt et al., 2017; Vincent et al., 2018). Initially, practitioner’s needs and decision requirements may be ill-defined, but become refined through an iterative process. Furthermore, coastal decisions are often characterized by conflicting stakeholder interests (Hinkel et al., 2018), which require the elaboration of joint perceptions, objectives, etc. Finally, users also require methods for applying information, including learning opportunities and technical assistance to address coastal resilience challenges (Tribbia and Moser, 2008; Hirschfeld and Hill, 2022).

The different participants in Figure 5 have different roles to play within the co-production process. Physical scientists need to place confidence judgments on the various SLR products available (Mastrandrea et al., 2011). Not all of these are equally plausible and practitioners need to choose actionable products that are well supported in the science community (van de Wal et al., 2022; Lipscomb et al., 2024) and match their approaches to risk management (Hinkel et al., 2019). The role of the practitioners and decision-makers is to express their context and needs, to assess their risk management approach, and to consider what adaptation options are feasible. The boundary worker’s role is to act as a bridge and ensure that decision analysis methods and available SLR products are integrated in a meaningful way to address the practitioner’s needs.

#### Adaptive decision making

Adaptive Decision Making (ADM) has been highlighted in coastal and more widely in climate adaptation (Hewitt et al., 2017; Vincent et al., 2018; Lawrence et al., 2018). ADM divides decisions into stages, implements flexible measures today and then progressively implements upgrades while learning how SLR unfolds (Ranger et al., 2013). Dynamic Adaptive Policy Pathways is a widely established framework for developing sequences of adaptation actions -- adaptation pathways -- and ranking them via multi-criteria analysis (Haasnoot et al., 2013). Additional ADM methods, including real-option analysis or optimal control are available, which find optimal economic trade-offs between adaptation investment today, including the cost of flexible design, versus delayed implementation while more is learned about SLR (Völz and Hinkel, 2023a). This approach is especially suitable for costly and long-lasting coastal adaptation decisions (e.g., dikes, surge barriers, land-use planning) (e.g., Woodward et al., 2014). Importantly, these approaches provide a framework for building adaptation measures iteratively, reducing the risk of maladaptation.

ADM frameworks are facilitated by a new class of SLR information which Hinkel et al (2019) termed learning scenarios. These estimate what additional information will be known at any given moment in the future about SLR beyond this moment (e.g., 2050 to 2100). They can be seen as a generalization of “normal” scenarios which provide information about future climate seen from a base year (i.e., today) and future moments in time. Within the GC, SLR learning scenarios based on IPCC AR6 scenarios were developed for the first time (Völz and Hinkel, 2023b) and applied to an economic assessment of adaptation pathways (Völz et al., 2024). Further practical research and implementation is required to fully explore the potential of ADM in coastal adaptation.

## Future priorities

This article has highlighted progress on SL science and its use in adaptation over the past decade including activities fostered by the WCRP’s GC. These are summarised and research priorities are identified as the IPCC AR7 cycle gets underway.

Regarding observations, global sea-level data derived from satellite altimeters are now of sufficient length to provide evidence of accelerating SLR. New radar altimeter instruments are providing higher resolution sea-level observations in the coastal zone. Together with measurements of ocean volume change (temperature and salinity) and mass change (changes in earth gravity), the SLR budget has been closed, including at regional scale. Sustainment of satellite-based ocean observations into the future will be crucial to the ongoing monitoring of sea-level change including at the coastline, where additional forcing factors (local sea levels, waves, river flows and vertical land movement) interact with SLR to drive extremes. Ongoing curation of global tide datasets will enable monitoring and attribution of extreme sea-level change. The development of reliable global vertical land movement data from analysis of tide gauges, GNSS and In-SAR satellite data is a key future priority at the coast, particularly in urban areas where the rate of subsidence may be many times the rate of climate-induced SLR.

Methodological advances in the development of SLR projections occurred within the IPCC AR6 cycle, including the use of physical emulators to derive SLR projections consistent with the AR6 assessment of climate sensitivity, which could be extended on the component level. Ice sheet and glacier models for estimating the mass contribution to SLR have been improved by the advent of model intercomparison projects. These more comprehensive approaches yield likely ranges of SLR that are broadly similar to previous assessments, but low-likelihood, high-end projections differ widely. Ice sheet and surface mass balance models that contribute to SLR projections have improved with more dynamic processes associated with ice sheet disintegration being developed. Work must continue however, to improve the agreement of ice sheet models to recent observations and to include other feedbacks between the ice and the rest of the climate system. Sea-level information on paleo time scales remains an important data source to constrain these models and future advances will help narrow uncertainties in long term and high-end projections.

Modelling the processes that contribute to extreme sea levels, including regional SLR, waves, tides and storm surges at global scales has advanced considerably over the past decade. Ongoing work is required to better represent small scale and relatively low frequency phenomena such as tropical cyclones in historical and future climate contexts. At the local coastal scale, model-based coastal assessments that integrate multiple oceanic and terrestrial (e.g. river runoff) factors and capture non-linear interactions and compound hazards remain a challenge, which will require further development and adoption of machine learning methods to increase the tractability of the problem. It is also vital to consider compound flooding when assessing and designing flood management.

The GC provided a forum to establish collaborative networks within the practitioner community to provide sustained peer support and learning. This was achieved through a first-ever global survey on sea-level information used by practitioners and their needs, a series of regional workshops which deepened understanding of practitioners needs in different regional contexts, and a SL conference that provided a dialogue between practitioners and researchers. These activities have highlighted various ongoing needs. Coastal climate services that enable the co-production of SLR projections with practitioners that build upon IPCC reports is essential. This includes the interpretation of global scale (IPCC) projections, particularly at the high end, and operational services in underrepresented areas such as the global south, small islands and deltas. Informational needs include localized sea-level and related hazard products, including decadal variability in near term projections and SLR projections across the full range of plausible emissions beyond 2100. Crucial to bridging between the science and practitioner communities is the role of boundary scientists working between both communities to translate and contextualise sea-level science using clearly defined criteria to support adaptation action. More effort to refine these criteria and activities to co-produce successful outcomes remains a priority.

Understanding and projecting SLR and its associated hazards is a multidisciplinary science spanning many physical and social science topics. To ensure that progress on the key challenges raised in this perspective continues in a timely and efficient manner, it will be critical to build functional, durable partnerships bridging science and society to ensure strong coordination of global SLR activities through the WCRP and other institutions.

To be submitted to ‘Cambridge Prisms: Coastal Futures’ describing the WCRP Grand Challenge on Regional Sea Level Change and its Impacts, including the Singapore Conference in 2022.

The authors declare no competing interests in the preparation of this manuscript.

## Supporting information

McInnes et al. supplementary material McInnes et al. supplementary material

## Supplementary material

The supplementary material for this article can be found at http://doi.org/10.1017/cft.2024.15.
