# Supplementary material for: Perspective on Regional Sea-level Change and Coastal Impacts
Source: Camb Prism Coast Futur. 2024 Nov 12;2:e16. doi: 10.1017/cft.2024.15 (PMC12337607; doi:10.1017/cft.2024.15)
Supplement: McInnes et al. supplementary material [file S2754720524000155sup001.pdf]

Supplementary information McInnes et al.,

Perspective on Regional Sea-level Rise and Coastal Impacts

Table S1: Sources and assumptions used for the high-end estimates shown in Figure 4

Source	Baseline	Median value (m)	Median value description	High-end value (m)	High-end value description
National Research Council (2012) (Darylmple, et al 2012)	2000	0.91	Median projection used 2007 IPCC SRES A1B projections from AR4 using Pardaens et al (2010).	1.40	The high-end steric contribution was determined by scaling the A1B projections for 2100 to high value of SRES A1FI. The cryospheric projection was an extrapolation from recent observed changes, ±1 standard deviation and including an additional dynamic contribution at the high end that increases ice discharge for GIS and AIS (Appendix E of report).
Third National Climate Assessment (2014) (Melillo et al 2014)	1992 (mid-point of the NTDE calculated from TG obs from 1983-2001)	-	Median projection not provided in NCA or Parris et al 2013, source document for SLR chapter.	2.00	High end derived from combination of ocean warming from IPCC AR4 SLR projections and maximum possible glacier and ice sheet loss by 2100 from Pfeffer et al., (2008) as per Parris et al (2013)
IPCC AR5 (Church et al., 2013)	1986-2005	0.74	Median of 5-95% projections for RCP 8.5.	-	No high end shown for reasons of unresolvable ambiguity
Horton et al. (2014)	2000	0.92	Median not shown in Horton et al 2014 but median for RCP 8.5 from Horton et al 2014 is taken from Horton et al 2020.	1.50	High end: 95th percentile projection for RCP 8.5 from expert survey
Sweet et al. (2017)	2000		No median figure was provided, only a median range of 0.5-1.3 m using sources supplementary to those of AR5 (p. 13)	2.50	High end: Authors attributed the high-end projection of 2.5 m ("extreme" scenario) to multiple sources: Sriver et al., 2012; Bamber and Aspinall, 2013; Miller et al., 2013; Rohling et al., 2013; Jevrejeva et al., 2014; Grinsted et al., 2015; Jackson and Jevrejeva, 2016; Kopp et al., 2014; DeConto and Pollard 2016). Analysis of these (Lipscomb et al 2024 in review) indicates primary reliance on Kopp et al 2014, which draws from Bamber and Aspinall 2013 for high end projections, as well as DeConto and Pollard 2016).

UKCP18 (Palmer et al., 2018)	1981-2000	0.78	These scenarios are derived from the AR5 scenarios (Church et al., 2013) for UK application. The global scenarios are updated to reflect larger Antarctic contributions following Levermann et al, (2014). The RCP8.5 global median rise to 2100 is 0.78m.	1.93	High end: Uses the upper end of H++ derived from UKCP09 (Lowe et al., 2009). A range of high-end sea-level increases termed H++ are given from present day (1980–1999) to 2095 for H++, but no time series is presented. The H++ scenarios were created as some potential users of UKCP09 requested a high-end coastal flooding scenario that lies above the best estimates of uncertainty for 21st century sea level rise and storm surges. Two lines of evidence were used to derive these ranges based on palaeo records (Rohling et al., 2008) and dynamic arguments (Pfeffer et al., 2008). The H++ range is intended to provide users with estimates of SLR within physical plausibility. While a likelihood was not assessed it was noted that the upper end of H++ is very unlikely to occur by 2100 and the main application of this scenario was envisaged as vulnerability testing. (see also Nicholls et al., 2014).
IPCC SROCC 2019 (Oppenheimer et al (2019)	1986-2005	0.84	Median RCP 8.5 estimate matching AR5 but with increased contribution from Antarctica from post-AR5 literature	2.00	Derived from Bamber et al 2019 Structured Expert Judgment
Horton et al. (2020)	1986-2005	0.93	Median taken from survey responses for 5-95 percentile range under RCP 8.5.	1.65	95th percentile of range developed from expert survey.
IPCC AR6 medium confidence (Fox-Kemper et al., 2021)	1995-2014	0.77	SSP5-8.5 median for medium confidence.	1.60	High-end is 95th percentile for RCP 8.5 with medium confidence
IPCC AR6 low confidence (Fox-Kemper et al., 2021)	1995-2014	1.00	Low confidence medians for SSP5-8.5 are shown in AR6 for MICI (0.99 m) and SEJ (1.0 m). We use the 1.0 figure here.	2.40	95th percentile of range for RCP 8.5 developed from Bamber et al 2019 Structured Expert Judgement, DeConto and Pollard 2016 and DeConto et al 2021.
Sweet et al. (2022)	2000	0.70	Median is for GMSL under 4°C warming, 2081-2100.	2.00	High end scenario of 2.0 meters GMSL reflects over 80% influence from the simulations combining AR6 low confidence projections based on Bamber et al 2019 and DeConto et al 2021 under high emissions.
Van de Wal et al. (2022)	1995-2014		Not estimated	1.55	High-end of range for 5°C warming by 2100 assuming SLR contributions are perfectly correlated

References

Bamber JL and Aspinall W (2013) An expert judgement assessment of future sea level rise from the ice sheets. *Nature Climate Change* **3**(4)**,** 424-427.

Bamber JL, Oppenheimer M, Kopp RE, Aspinall WP and Cooke RM (2019) Ice sheet contributions to future sea-level rise from structured expert judgment. *Proceedings of the National Academy of Sciences* **116**(23)**,** 11195-11200. https://doi.org/10.1073/pnas.1817205116.

Church JA, Clark PU, Cazenave A, Gregory JM, Jevrejeva S, Levermann A, Merrifield MA, Milne GA, Nerem RS and Nunn PD (2013) Sea level change. In: *Climate Change 2013: The Physical Science Basis. Contribution of Working Group I to the Fifth Assessment Report of the Intergovernmental Panel on Climate Change* [Stocker, T.F., D. Qin, G.-K. Plattner, M. Tignor, S.K. Allen, J. Boschung, A. Nauels, Y. Xia, V. Bex and P.M. Midgley (eds.)]. Cambridge University Press, Cambridge, United Kingdom and New York, NY, USA. PM Cambridge University Press.

Dalrymple RA, Breaker LC, Brooks BA, Cayan DR, Griggs GB, Han W, Horton BP, Hulbe CL, McWilliams JC, Mote PW, Pfeffer WT, Reed DJ and Shum CK (2012) Sea-Level Rise for the Coasts of California, Oregon, and Washington: Past, Present, and Future. . National Research Council.

DeConto RM and Pollard D (2016) Contribution of Antarctica to past and future sea-level rise. *Nature* **531,** 591. https://doi.org/10.1038/nature17145 https://www.nature.com/articles/nature17145#supplementary-information.

DeConto RM, Pollard D, Alley RB, Velicogna I, Gasson E, Gomez N, Sadai S, Condron A, Gilford DM, Ashe EL, Kopp RE, Li D and Dutton A (2021) The Paris Climate Agreement and future sea-level rise from Antarctica. *Nature* **593**(7857)**,** 83-89. https://doi.org/10.1038/s41586-021-03427-0.

Fox-Kemper B, Hewitt HT, Xiao C, Aðalgeirsdóttir G, Drijfhout SS, Edwards TL, Golledge NR, Hemer M, Kopp RE, Krinner G, Mix A, Notz D, Nowicki S, Nurhati IS, Ruiz L, Sallée J-B, Slangen ABA and Yu Y (2021) Ocean, Cryosphere and Sea Level Change. In Masson-Delmotte V, P. Zhai, A. Pirani, S.L. Connors, C. Péan, S. Berger, N. Caud, Y. Chen, L. Goldfarb, M.I. Gomis, M. Huang, K. Leitzell, E. Lonnoy, J.B.R. Matthews, T.K. Maycock, T. Waterfield, O. Yelekçi, R. Yu, and B. Zhou (ed), *Climate Change 2021: The Physical Science Basis. Contribution of Working Group I to the Sixth Assessment Report of the Intergovernmental Panel on Climate Change.* Cambridge University Press, Cambridge, United Kingdom and New York, NY, USA, 1211–1362.

Grinsted A, Jevrejeva S, Riva RE and Dahl-Jensen D (2015) Sea level rise projections for northern Europe under RCP8. 5. *Climate Research* **64**(1)**,** 15-23.

Horton BP, Khan NS, Cahill N, Lee JSH, Shaw TA, Garner AJ, Kemp AC, Engelhart SE and Rahmstorf S (2020) Estimating global mean sea-level rise and its uncertainties by 2100 and 2300 from an expert survey. *npj Climate and Atmospheric Science* **3**(1)**,** 18. https://doi.org/10.1038/s41612-020-0121-5.

Horton BP, Rahmstorf S, Engelhart SE and Kemp AC (2014) Expert assessment of sea-level rise by AD 2100 and AD 2300. *Quaternary Science Reviews* **84,** 1-6. https://doi.org/https://doi.org/10.1016/j.quascirev.2013.11.002.

Jackson LP and Jevrejeva S (2016) A probabilistic approach to 21st century regional sea-level projections using RCP and High-end scenarios. *Global and Planetary Change* **146,** 179-189.

Jevrejeva S, Grinsted A and Moore JC (2014) Upper limit for sea level projections by 2100. *Environmental Research Letters* **9**(10)**,** 104008.

Kopp RE, Horton BP, Kemp AC and Tebaldi C (2014) Past and future sea-level rise along the coast of North Carolina, United States. *arXiv preprint arXiv:1410.8369*.

Levermann A, Winkelmann R, Nowicki S, Fastook JL, Frieler K, Greve R, Hellmer HH, Martin MA, Meinshausen M and Mengel M (2014) Projecting Antarctic ice discharge using response functions from SeaRISE ice-sheet models. *Earth System Dynamics* **5**(2)**,** 271-293.

Lipscomb WH, Behar D and Morrison MA (2024) Brief communication: Sea-level projections, adaptation planning, and actionable science. *EGUsphere* (2024) [preprint], https://doi.org/10.5194/egusphere-2024-534**.**

Lowe J, Howard T, Pardaens A, Tinker J, Holt J, Wakelin S, Milne G, Leake J, Wolf J, Horsburgh K, Reeder T, Jenkins G, Ridley J, Dye S and Bradley S (2009) *UK Climate Projections science report: Marine and coastal projections*. Met Office Hadley Centre, Exeter, UK.

Melillo JM, Richmond TC and Yohe GWE (2014) Climate Change Impacts in the United States: The Third National Climate Assessment. U.S. Global Change Research Program.

Miller KG, Kopp RE, Horton BP, Browning JV and Kemp AC (2013) A geological perspective on sea-level rise and its impacts along the US mid-Atlantic coast. *Earth's Future* **1**(1)**,** 3-18.

Nicholls RJ, Hanson SE, Lowe JA, Warrick RA, Lu X and Long AJ (2014) Sea-level scenarios for evaluating coastal impacts. *Wiley Interdisciplinary Reviews: Climate Change* **5**(1)**,** 129-150.

Oppenheimer M, Glavovic B, Abd-Elgawad A, Cai R, Cifuentes-Jara M, Deconto R, Ghosh T, Hay J, Hinkel J, Isla F, A.K. M, Marzeion B, Meyssignac B, Sebesvari Z, Smit AJ and van de Wal RSW (2019) Sea Level Rise and Implications for Low Lying Islands, Coasts and Communities in In H.-O. Pörtner DCR, V. Masson-Delmotte, P. Zhai, M. Tignor, E. Poloczanska, K. Mintenbeck, A. Alegría, M. Nicolai, A. Okem, J. Petzold, B. Rama, N.M. Weyer (ed), *IPCC Special Report on Oceans and Cryosphere.* Cambridge University Press, Cambridge, UK and New York, NY, USA, 321-445.

Palmer M, Howard T, Tinker J, Lowe J, Bricheno L, Calvert D, Edwards T, Gregory J, Harris G, Krijnen J, Pickering M, Roberts C and Wolf J (2018) UKCP18 Marine report. Met Office Hadley Centre, Exeter, UK.

Pardaens AK, Gregory JM and Lowe J (2011) A model study of factors influencing projected changes in regional sea level over the twenty-first century. *Climate Dynamics* **36,** 2015-2033.

Parris A, Bromirski P, Burkett V, Cayan D, Culver M, Hall J, Horton R, Knuuti K, Moss R, Obeysekera J, Sallenger A and Weiss J (2012) Global Sea Level Rise Scenarios for the US National Climate Assessment. National Oceanic and Atmospheric Administration.

Pfeffer WT, Harper JT and O'Neel S (2008) Kinematic constraints on glacier contributions to 21st-century sea-level rise. *Science* **321**(5894)**,** 1340-1343.

Rohling EJ, Grant K, Hemleben C, Siddall M, Hoogakker B, Bolshaw M and Kucera M (2008) High rates of sea-level rise during the last interglacial period. *Nature geoscience* **1**(1)**,** 38-42.

Rohling EJ, Haigh ID, Foster GL, Roberts AP and Grant KM (2013) A geological perspective on potential future sea-level rise. *Scientific Reports* **3**(1)**,** 3461.

Sriver RL, Urban NM, Olson R and Keller K (2012) Toward a physically plausible upper bound of sea-level rise projections. *Climatic Change* **115,** 893-902.

Sweet WV, Hamlington BD, Kopp RE, Weaver CP, Barnard PL, Bekaert D, Brooks W, Craghan M, Dusek G, Frederikse T, Garner G, Genz AS, Krasting JP, Larour E, Marcy D, Marra JJ, Obeysekera J, Osler M, Pendleton M, Roman D, Schmied L, Veatch W, White KD and Zuzak C (2022) Global and Regional Sea Level Rise Scenarios for the United States: Updated Mean Projections and Extreme Water Level Probabilities Along U.S. Coastlines. NOAA. National Oceanic and Atmospheric Administration, National Ocean Service, Silver Spring, MD

Sweet WV, Kopp RE, Weaver CP, Obeysekera J, Horton BP, Thieler ER and Zervas C (2017) Global and Regional Sea Level Rise Scenarios for the United States. NOAA Technical Report NOS CO-OPS 083.

van de Wal RSW, Nicholls RJ, Behar D, McInnes K, Stammer D, Lowe JA, Church JA, DeConto R, Fettweis X, Goelzer H, Haasnoot M, Haigh ID, Hinkel J, Horton BP, James TS, Jenkins A, LeCozannet G, Levermann A, Lipscomb WH, Marzeion B, Pattyn F, Payne AJ, Pfeffer WT, Price SF, Seroussi H, Sun S, Veatch W and White K (2022) A High-End Estimate of Sea Level Rise for Practitioners. *Earth's Future* **10**(11)**,** e2022EF002751. https://doi.org/https://doi.org/10.1029/2022EF002751.
