# Supplementary material for: Perspective on Regional Sea-level Change and Coastal Impacts
Source: Camb Prism Coast Futur. 2024 Nov 12;2:e16. doi: 10.1017/cft.2024.15 (PMC12337607; doi:10.1017/cft.2024.15)
Supplement: Peer review history

# Editor report: Recommendation: Perspective on Regional Sea-level Change and Coastal Impacts — R0/PR2

**DOI:** 10.1017/cft.2024.15.pr2

**Affiliations:** School of Civil and Environmental Engineering, UNSW, Australia

Dear Dr. McInnes and co-authors,

I have read your paper and along with the review feel that the paper can be suitable for publication in Coastal Futures upon addressing some minor comments. These are provided in the attached review by R1. 

I also note that the paper exceeded the guidelines wrt length. I could not see specifics on what could be removed and R1 has actually asked for a bit more so I would encourage you to be succinct in any additions made at this time. 

All the best,

Kristen Splinter

Handling Editor, Cambridge Prisms: Coastal Futures

# Editor report: Decision: Perspective on Regional Sea-level Change and Coastal Impacts — R0/PR3

**DOI:** 10.1017/cft.2024.15.pr3

**Affiliations:** Geography, Cambridge University, United Kingdom of Great Britain and Northern Ireland

No accompanying comment.

# Editor report: Recommendation: Perspective on Regional Sea-level Change and Coastal Impacts — R1/PR5

**DOI:** 10.1017/cft.2024.15.pr5

**Affiliations:** School of Civil and Environmental Engineering, UNSW, Australia

I do believe the authors have sufficiently responded to the review comments in this revised version within the limits of the word count allowed.

# Editor report: Decision: Perspective on Regional Sea-level Change and Coastal Impacts — R1/PR6

**DOI:** 10.1017/cft.2024.15.pr6

**Affiliations:** Geography, Cambridge University, United Kingdom of Great Britain and Northern Ireland

No accompanying comment.
